# Supplementary material for: The deubiquitinase Rpn11 functions as an allosteric ubiquitin sensor to promote substrate engagement by the 26S proteasome
Source: Cell Rep. Author manuscript; Available in PMC 2025 Jul 13. (PMC12256112; doi:10.1016/j.celrep.2025.115736)
Supplement: 1 [file NIHMS2092638-supplement-1.pdf]

Cell Reports, Volume *44*

Supplemental information

The deubiquitinase Rpn11 functions as an allosteric ubiquitin sensor to promote substrate engagement by the 26S proteasome

Zaw Min Htet, Ken C. Dong, and Andreas Martin

Supplemental Figures and Tables:

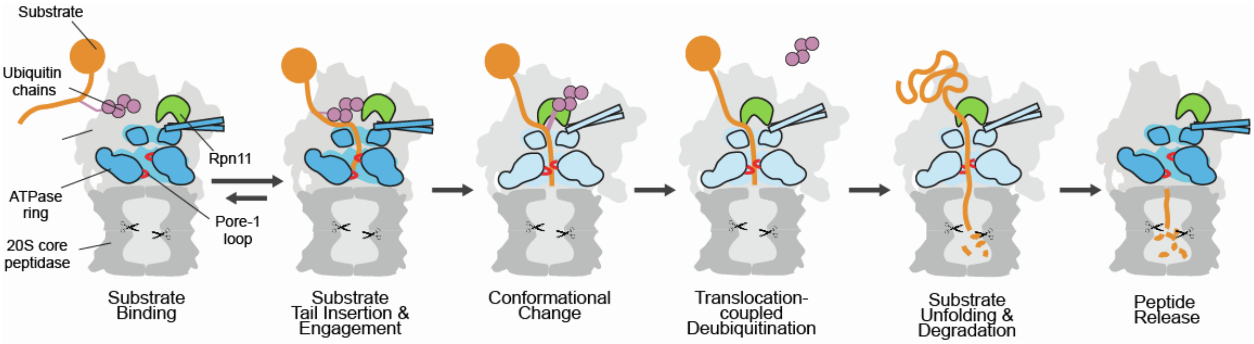

Supplemental Fig. 1: Current model for individual steps of substrate degradation by the 26S proteasome. Related to Figure 1. The cutaway view of the 26S proteasome shows the 20S core particle in dark grey, the lid and non-ATPase subunits of the base subcomplex in light grey, the ATPase hexamer in blue, translocating pore-1 loops in red, the Rpn11 deubiquitinase in green, the substrate in orange, and the substrate-attached poly-ubiquitin chain in purple. After a substrate is recruited through ubiquitin binding to a proteasomal receptor, the substrate's flexible initiation region diffuses into the central channel of the ATPase ring. Upon successful substrate engagement with the pore-1 loops, the base ATPase transitions from the engagement-competent s1 state (dark blue) to processing-competent non-s1 states (light blue) for substrate unfolding and processive translocation into the core particle for proteolytic cleavage. Ubiquitin chains are removed from the substrate by Rpn11 in a co-translocational manner. After complete threading of the substrate polypeptide, the base ATPase switches back to the engagement-competent s1 state.

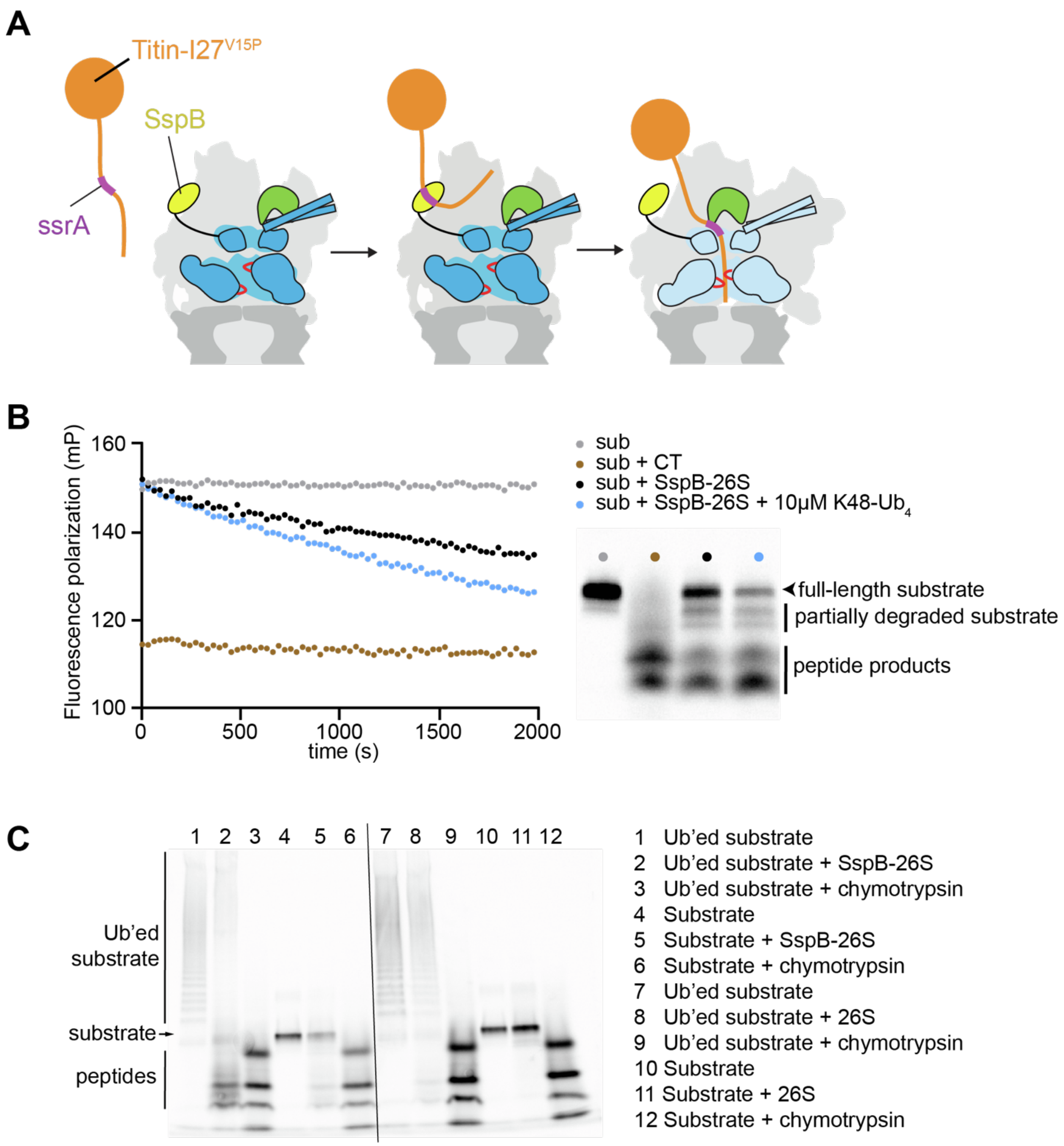

Supplemental Fig. 2: Ubiquitin-independent SspB-mediated substrate degradation by the 26S proteasome. Related to Figure 1. A) Schematic for the degradation of a titin^{V15P} model substrate (orange) with the ssrA degron sequence (purple) in the C-terminal unstructured initiation region. The engineered proteasome variant contains the ssrA-binding SspB adaptor (yellow) from

E. coli fused to the N-terminus of the Rpt2 ATPase subunit of the base subcomplex (blue). B) Left: Example traces for the degradation of the ssrA-tagged titin^{V15P} model substrate by SspB-fused 26S proteasome in the absence or presence of unanchored K48-linked tetra-ubiquitin chains (K48-Ub_4). The substrate was N-terminally labeled with fluoresceine amidite (FAM) to monitor degradation by fluorescence polarization. Also shown is the complete cleavage by chymotrypsin as a control. Right: SDS-PAGE analysis of aliquots taken after 60 min from the polarization-monitored degradation samples. C) SDS-PAGE (4 - 20%) analysis of the endpoints for the degradation of ubiquitinated (Ub'ed) or unmodified ssrA-containing titin^{V15P} substrate by SspB-fused or wild-type 26S proteasome or chymotrypsin.

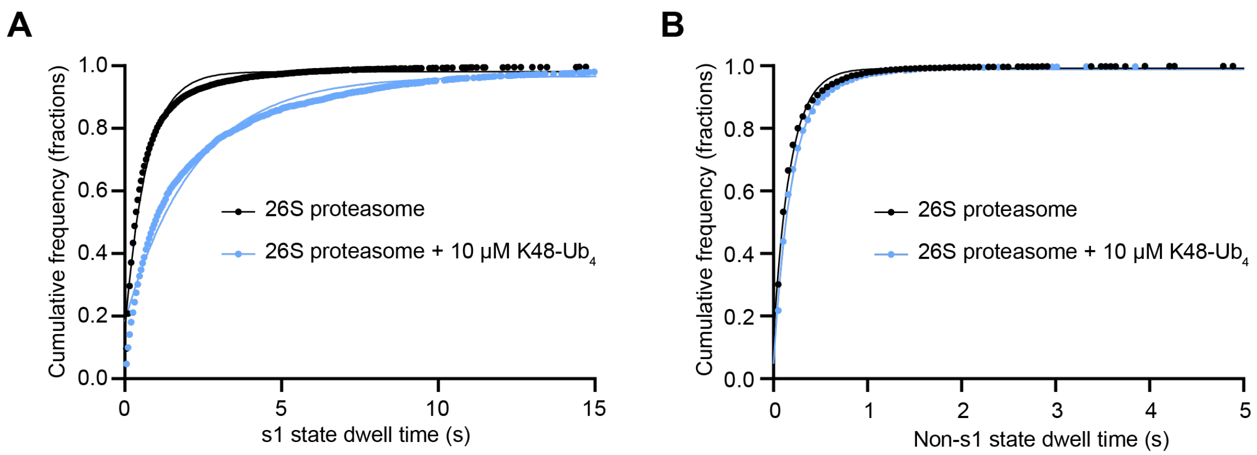

Supplemental Fig. 3: Determination of the transition rates of the proteasome conformations. Related to Figure 1. Cumulative frequencies for the dwell-time distributions of the low-FRET s1 state (A) and the high-FRET non-s1 states (B) during the conformational switching of the substrate-free wild-type proteasome in the absence of K48-Ub_4 (black, N = 9297 events for s1 state, N = 9289 events for non-s1 states) and in the presence of K48-Ub_4 (blue, N = 3168 events for s1 state, N = 3161 events for non-s1 states), with fits to single exponentials shown as black and blue lines.

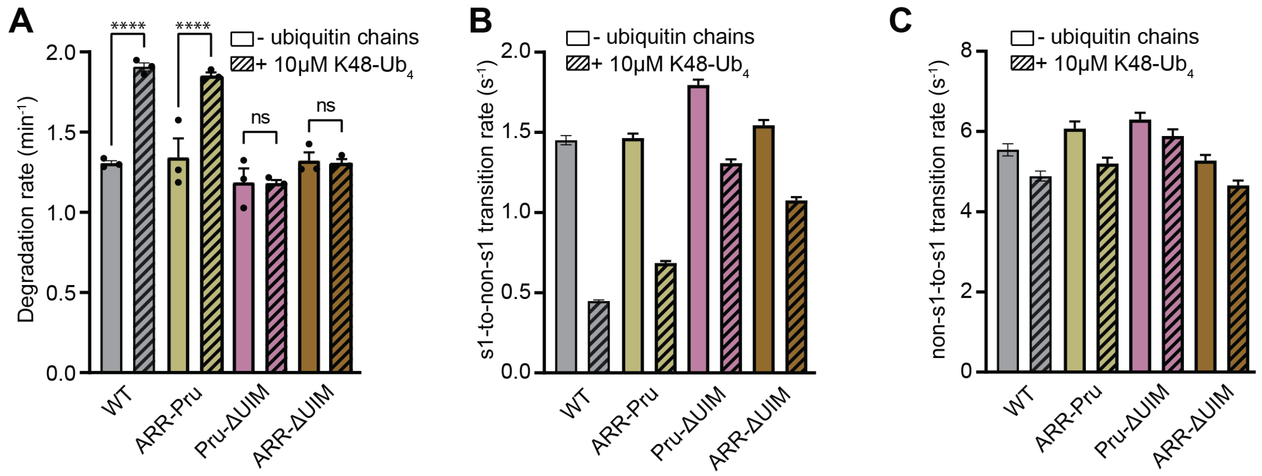

Supplemental Fig. 4: The ubiquitin interacting motif of Rpn10 mediates the allosteric effect of ubiquitin chains on the proteasomal substrate degradation and conformational dynamics. Related to Figure 1. A) Unanchored K48-Ub$_4$ ubiquitin-chain dependent stimulation of SspB-delivered substrates degradation by wild-type and receptor-deficient 26S proteasomes, carrying a combination of mutations in the T1 site of Rpn1 (ARR), mutations in the Pru domain of Rpn13 (Pru), or a deletion of Rpn10's UIM (ΔUIM). Shown are the averages of three technical replicates with error bars representing the standard errors of mean. Statistical significance was calculated using an ordinary one-way ANOVA test. ns $p>0.9999$, **** $p<0.0001$. B) Effects of unanchored K48-Ub$_4$ on the s1-to-non-s1-transition rates for wild-type and receptor-deficient proteasomes with a combination of ARR, Pru, or ΔUIM mutations. Shown are the transition rates calculated by fitting the s1-state dwell time distribution of > 2900 transition events observed in at least 200 FRET-efficiency traces from two technical replicates of the proteasome conformational dynamics assay, with error bars representing the standard errors of the fit. C) Effects of unanchored K48-Ub$_4$ on the non-s1-to-s1 transition rates for wild-type and receptor-deficient proteasomes with a combination of ARR, Pru, or ΔUIM mutations Shown are the transition rates calculated by fitting the non-s1-state dwell time distribution of > 2900 transition events observed in at least 200 FRET-efficiency traces from two technical replicates of the proteasome conformational dynamics assay, with error bars representing the standard errors of the fit.

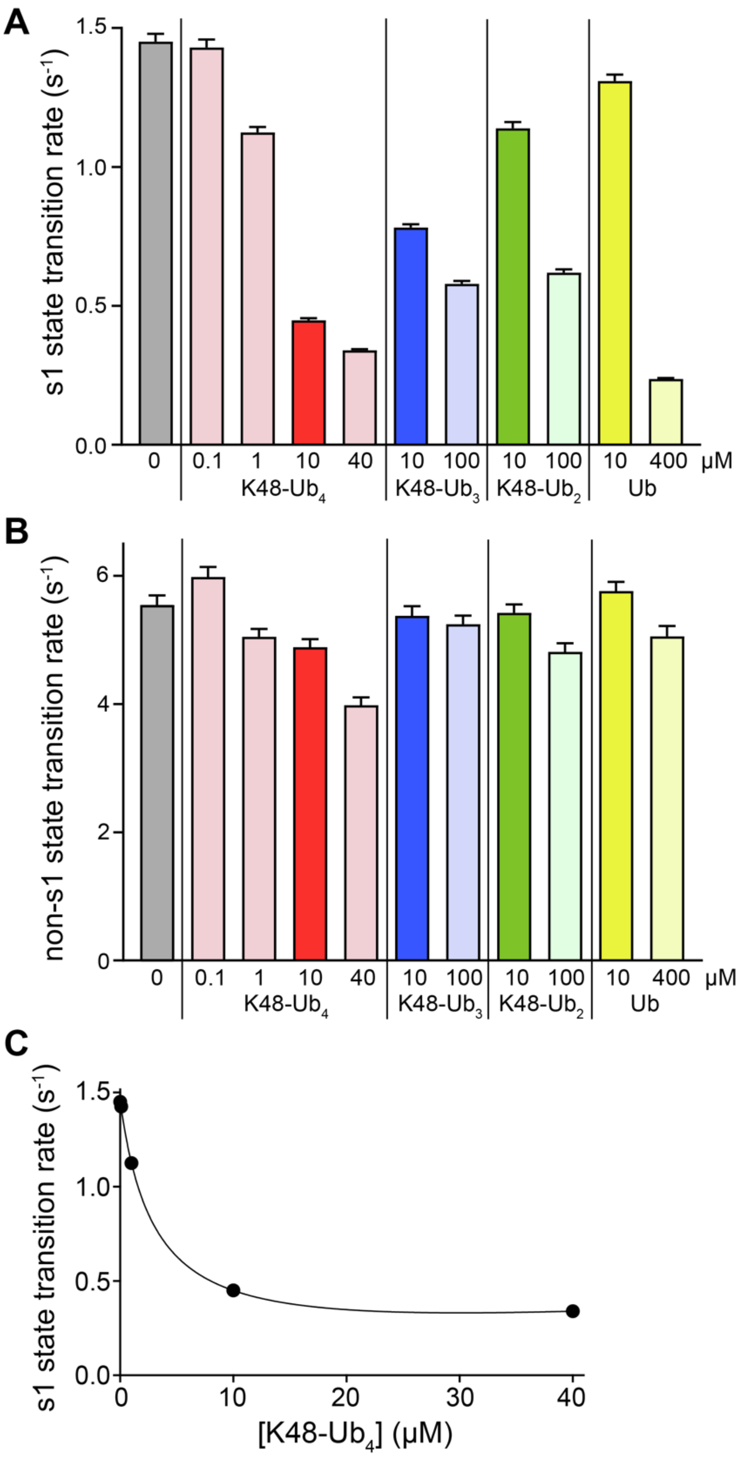

Supplemental Fig. 5: Ubiquitin chains of various lengths and even mono-ubiquitin affect proteasome conformational dynamics. Related to Figure 1. A) Effects of unanchored K48-linked ubiquitin tetramer (K48-Ub_4), trimer (K48-Ub_3), dimer (K48-Ub_2), and mono-ubiquitin (Ub) at different concentrations on the s1-to-non-s1-transition rate of wild-type proteasome. Shown are

the transition rates calculated by fitting the s1-state dwell time distribution of > 2900 transition events observed in at least 200 FRET-efficiency traces from two technical replicates of the proteasome conformational dynamics assay, with error bars representing the standard errors of the fit. B) Effects of unanchored K48-Ub_4, K48-Ub_3, K48-Ub_2, and Ub at different concentrations on the non-s1-to-s1-transition rate of wild-type proteasome. Shown are the transition rates calculated by fitting the non-s1-state dwell time distribution of > 2900 transition events observed in at least 200 FRET-efficiency traces from two technical replicates of the proteasome conformational dynamics assay, with error bars representing the standard errors of the fit. C) Binding curve for the interaction of unanchored K48-Ub_4 with wild-type proteasome based the allosteric effects on the s1-to-non-s1 conformational transition rate at different concentrations (data see panel A), revealing an apparent affinity of K_D ~ 3.1 μM.

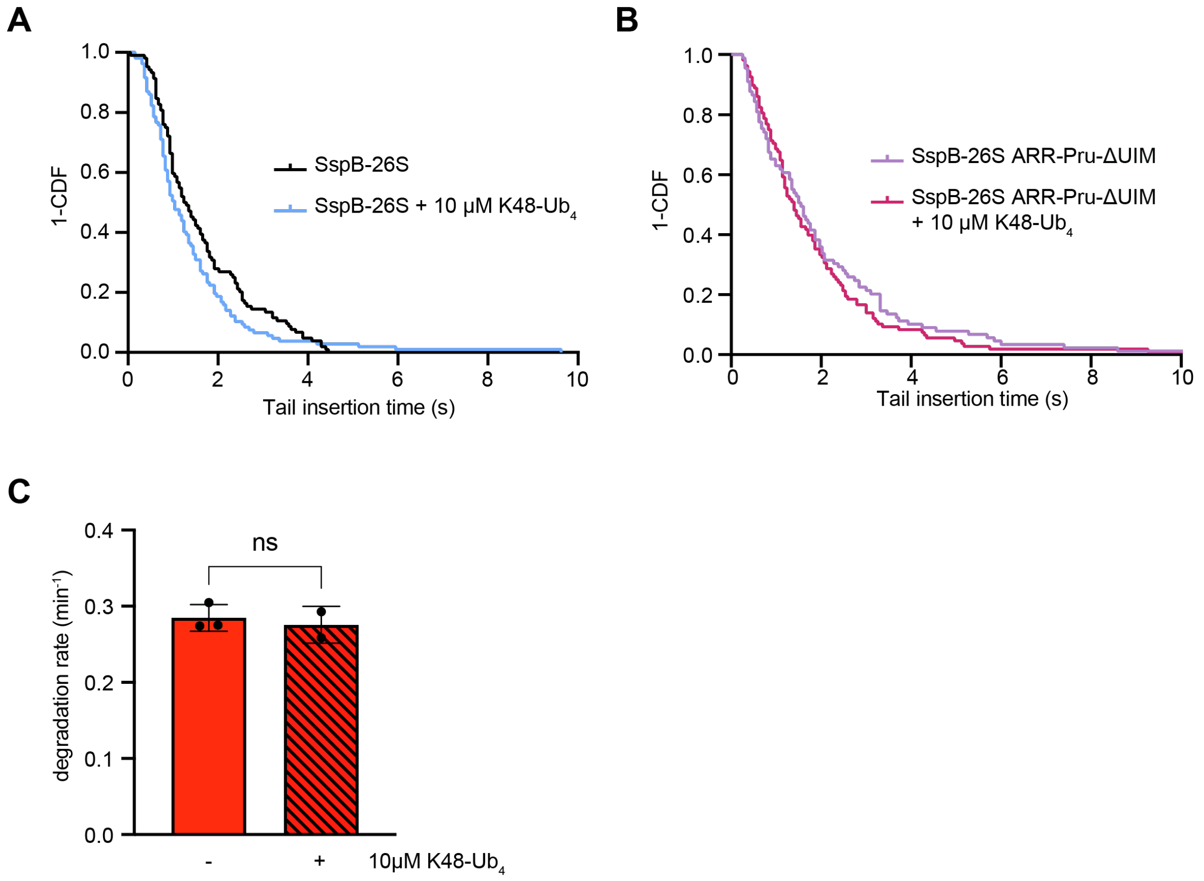

Supplemental Fig. 6: Effects of ubiquitin chains on substrate tail insertion and degradation kinetics. Related to Figure 4. A,B) Survival (1-CDF) plots for the tail-insertion times of SspB-delivered titin^{V15P} substrate and wild-type (A) or triple-receptor-deficient, ARR-Pru-ΔUIM-mutant proteasome (B) in the absence (black, N = 104 events; purple, N = 89 events) or the presence (blue, N = 107 events; magenta, N = 108 events) of K48-Ub_4 ubiquitin chains. Comparing the survival plot using the Gehan-Breslow-Wilcoxon test gives p-values of 0.0189 and 0.7842 for wild-type and triple-receptor-deficient, ARR-Pru-ΔUIM-mutant proteasome, respectively. C) Rates for the degradation of SspB-delivered GS substrate triple-receptor-deficient (ARR-Pru-ΔUIM) 26S proteasome in the absence and presence of K48-Ub_4 ubiquitin chains. Shown are the averages from 3 technical replicates, with the error bars indicating the standard error of mean. Statistical significance was calculated using an ordinary one-way ANOVA test. ns non-significant with $p = 0.6526$.

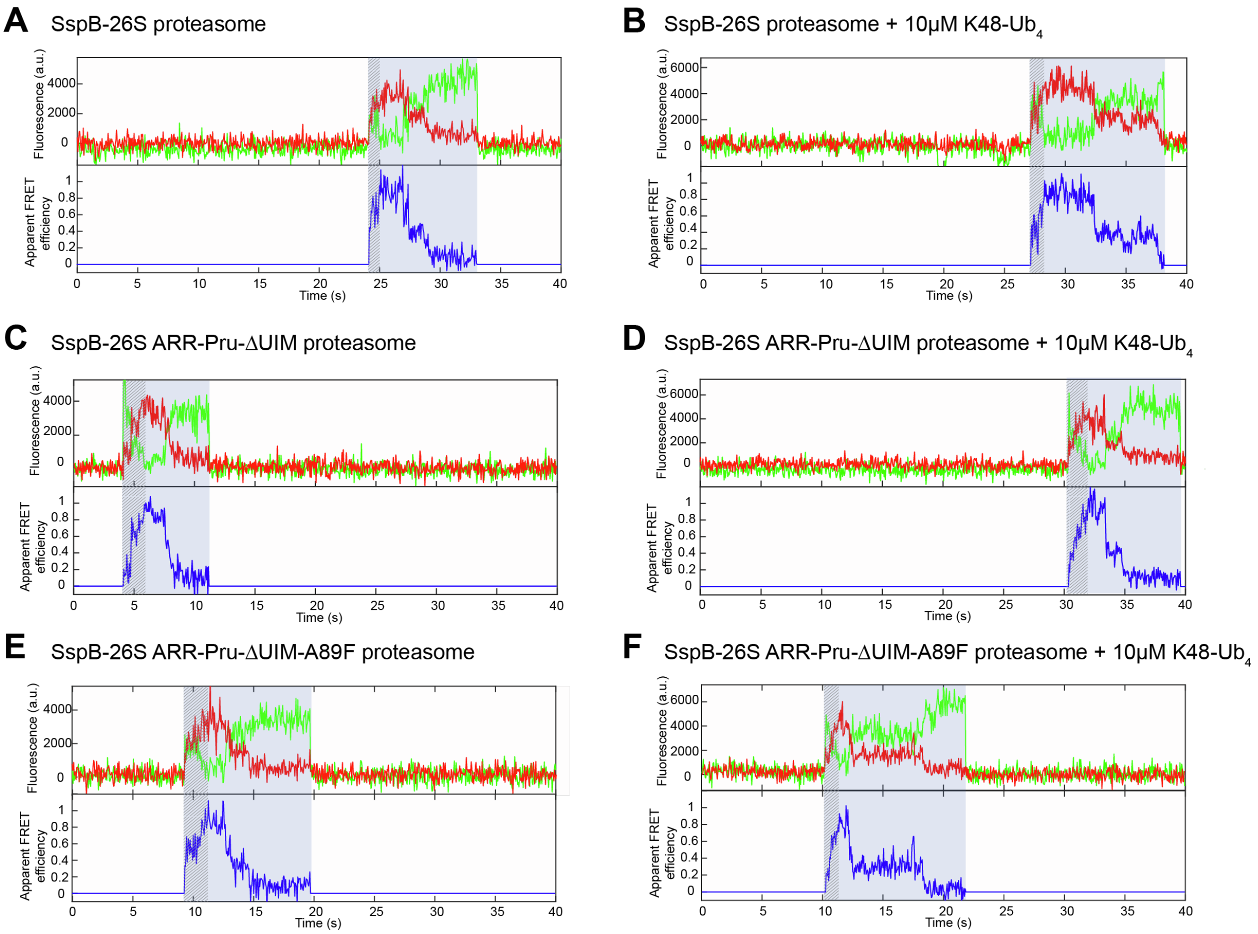

Supplemental Fig. 7: Representative traces for the single-molecule substrate-processing assay monitoring the degradation of the SspB-delivered titinV15P model substrate. Related to Figure 4. Substrate degradation by SspB-fused wild-type proteasome in the absence (A) and presence (B) of unanchored tetraubiquitin chains K48-Ub_4. Substrate degradation by SspB-fused triple-receptor-deficient (ARR-Pru-ΔUIM) proteasome in the absence (C) and presence (D) of unanchored tetraubiquitin chains K48-Ub_4. Substrate degradation by SspB-fused triple-receptor-deficient proteasome with Rpn11 A89F mutation ((ARR-Pru-ΔUIM-A89F) in the absence (E) and presence (F) of unanchored tetraubiquitin chains K48-Ub_4.

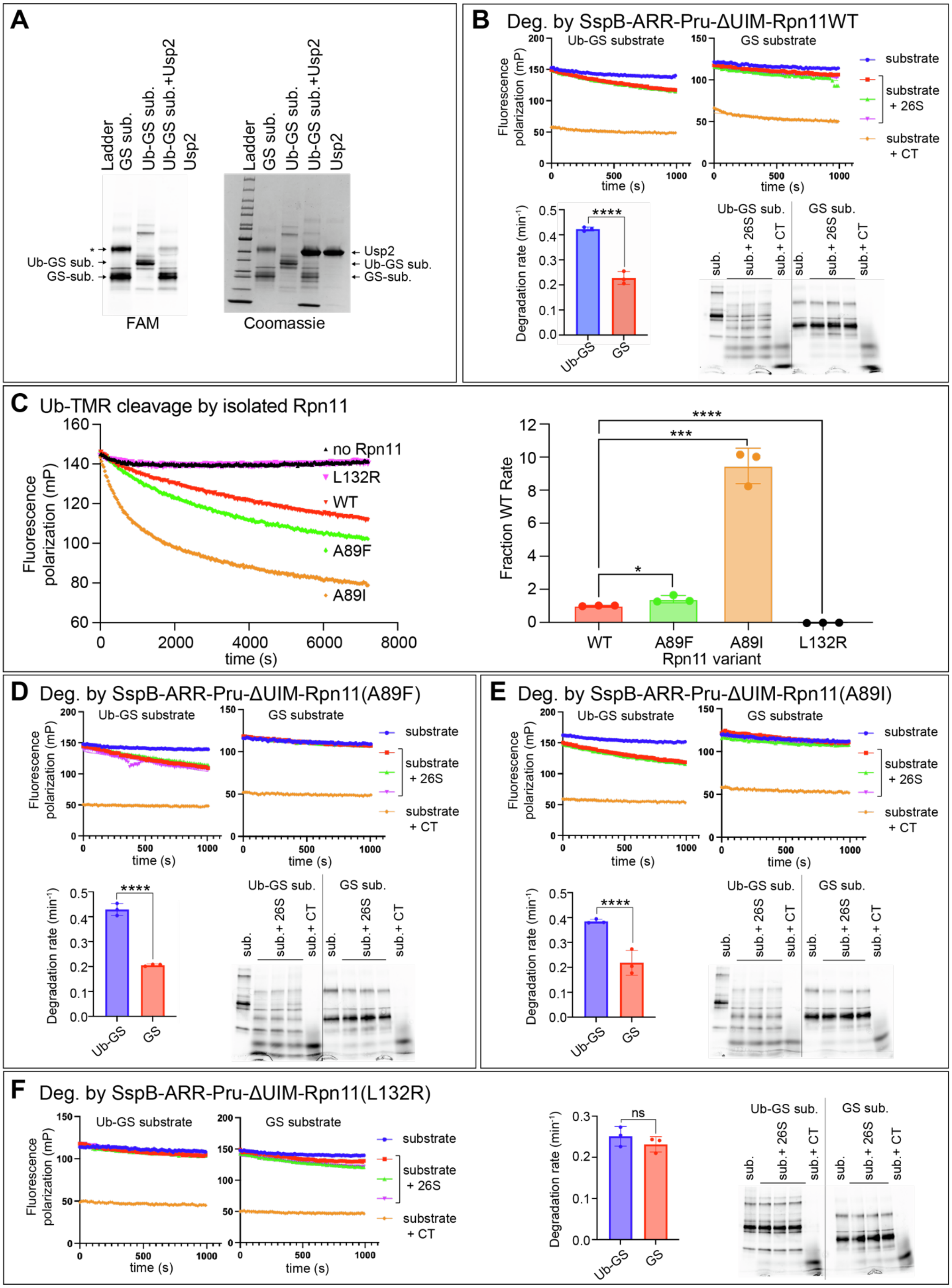

A
Ladder
GS sub.
Ub-GS sub.
Ub-GS sub.+Usp2
Usp2
Ub-GS sub.
GS-sub.
FAM
Coomassie
Usp2
Ub-GS sub.
GS-sub.
B Deg. by SspB-ARR-Pru-ΔUIM-Rpn11WT
Ub-GS substrate
GS substrate
Fluorescence polarization (mP)
time (s)
substrate
substrate + 26S
substrate + CT
Degradation rate (min⁻¹)
****
Ub-GS
GS
Ub-GS sub.
GS sub.
sub.
sub.+ 26S
sub.+ CT
C Ub-TMR cleavage by isolated Rpn11
no Rpn11
L132R
WT
A89F
A89I
Fluorescence polarization (mP)
time (s)
Fraction WT Rate
Rpn11 variant
****
***
*
D Deg. by SspB-ARR-Pru-ΔUIM-Rpn11(A89F)
E Deg. by SspB-ARR-Pru-ΔUIM-Rpn11(A89I)
F Deg. by SspB-ARR-Pru-ΔUIM-Rpn11(L132R)
ns

Supplemental Fig. 8: Characterization of Rpn11-mutant effects on de-ubiquitination activity and GS-substrate degradation. Related to Figure 5. A) Fluorescence scan (left) and Coomassie stain of a 4-20% SDS-PAGE gel showing the fluoresceine (FAM) labeled non-ubiquitinated GS-substrate and ubiquitinated Ub-GS-substrate before and after treatment with the deubiquitinase Usp2. * marks a dimeric artifact of the GS-substrate. B) SspB/ssrA-mediated degradation of the GS-substrate and Ub-GS substrate by the ubiquitin-receptor-less, SspB-fused 26S proteasome. Top: Example traces for the fluorescence polarization changes of the Ub-GS and GS substrates alone, during multiple-turnover proteasomal degradation, or in the presence of chymotrypsin (CT). Bottom left: calculated degradation rates. Shown are the averages of three technical replicates with error bars representing the standard errors of the mean. Statistical significance was calculated using an ordinary one-way ANOVA test. **** $p < 0.0001$. Bottom right: Coomassie-stained SDS-PAGE (4-20%) analysis of the degradation end points. C) Cleavage of Ubiquitin-TAMRA by isolated Rpn11/Rpn8 and its variants. Left: representative fluorescence-polarization traces for the cleavage of Ub-TAMRA by wild-type Rpn11 and its mutants. Right: Calculated relative rates for the deubiquitination activities of wild-type and mutant Rpn11. Shown are the averages of three technical replicates with error bars representing the standard errors of the mean. Statistical significance was calculated using an unpaired t-test. * $p = 0.0348$, *** $p = 0.0002$, **** $p <0.0001$. D) SspB/ssrA-mediated degradation of the GS-substrate and Ub-GS substrate by the ubiquitin-receptor-less, SspB-fused 26S proteasome with Rpn11 A89F mutation. Top: Example traces for the fluorescence polarization changes of the Ub-GS and GS substrates alone, during multiple-turnover proteasomal degradation, or in the presence of chymotrypsin (CT). Bottom left: calculated degradation rates. Shown are the averages of three technical replicates with error bars representing the standard errors of the mean. Statistical significance was calculated using an ordinary one-way ANOVA test. **** $p < 0.0001$. Bottom right: Coomassie-stained SDS-PAGE (4-20%) analysis of the degradation end points. E) SspB/ssrA-mediated degradation of the GS-substrate and Ub-GS substrate by the ubiquitin-receptor-less, SspB-fused

26S proteasome with Rpn11 A89I mutation. Top: Example traces for the fluorescence polarization changes of the Ub-GS and GS substrates alone, during multiple-turnover proteasomal degradation, or in the presence of chymotrypsin (CT). Bottom left: calculated degradation rates. Shown are the averages of three technical replicates with error bars representing the standard errors of the mean. Statistical significance was calculated using an ordinary one-way ANOVA test. **** $p < 0.0001$. Bottom right: Coomassie-stained SDS-PAGE (4-20%) analysis of the degradation end points. F) SspB/ssrA-mediated degradation of the GS-substrate and Ub-GS substrate by the ubiquitin-receptor-less, SspB-fused 26S proteasome with Rpn11 L132R mutation. Left: Example traces for the fluorescence polarization changes of the Ub-GS and GS substrates alone, during multiple-turnover proteasomal degradation, or in the presence of chymotrypsin (CT). Right: calculated degradation rates and Coomassie-stained SDS-PAGE (4-20%) analysis of the degradation end points. For the degradation rates, shown are the averages of three technical replicates with error bars representing the standard errors of the mean. Statistical significance was calculated using an ordinary one-way ANOVA test. ns non-significant with $p = 0.8005$.

Supplemental Table 1:

Proteasome variant	k_{s1} (s^{-1})	$k_{non\text{-}s1}$ (s^{-1})
WT proteasome	1.45 ± 0.03	5.54 ± 0.15
WT proteasome + 10 μM K48-Ub_4	0.45 ± 0.01	4.89 ± 0.13
ΔUIM proteasome	1.64 ± 0.03	5.54 ± 0.15
ΔUIM proteasome + 10 μM K48-Ub_4	1.29 ± 0.03	5.01 ± 0.11
ARR proteasome	1.28 ± 0.03	5.07 ± 0.14
ARR proteasome + 10 μM K48-Ub_4	0.58 ± 0.01	4.16 ± 0.10
Pru proteasome	1.67 ± 0.03	6.44 ± 0.17
Pru proteasome + 10 μM K48-Ub_4	0.62 ± 0.01	5.13 ± 0.14
ARR-Pru proteasome	1.46 ± 0.03	6.08 ± 0.18
ARR-Pru proteasome + 10 μM K48-Ub_4	0.69 ± 0.01	5.20 ± 0.15
Pru-ΔUIM proteasome	1.80 ± 0.03	6.30 ± 0.16
Pru-ΔUIM proteasome + 10 μM K48-Ub_4	1.31 ± 0.02	5.89 ± 0.17
ARR-ΔUIM proteasome	1.54 ± 0.03	5.27 ± 0.14
ARR-ΔUIM proteasome + 10 μM K48-Ub_4	1.08 ± 0.02	4.65 ± 0.12
ARR-Pru-ΔUIM proteasome	1.47 ± 0.03	5.06 ± 0.14
ARR-Pru-ΔUIM proteasome + 10 μM K48-Ub_4	1.14 ± 0.02	4.58 ± 0.10
$Rpn10^{EKK}$ proteasome	0.87 ± 0.01	6.61 ± 0.20
ARR-Pru-ΔUIM-A89F proteasome	1.40 ± 0.03	5.32 ± 0.13
ARR-Pru-ΔUIM-A89F proteasome + 10 μM K48-Ub_4	0.54 ± 0.01	4.22 ± 0.10

Supplemental Table 1: Rates of proteasome conformational switching. Rates for the conformational switching from the s1 to non-s1 states (k_{s1}) and from non-s1 to s1 states ($k_{non\text{-}s1}$) were determined by Hidden-Markov-modeling of the results from the FRET-based conformational dynamic assay for wild-type proteasome and various proteasome variants in the absence and presence of unanchored K48-linked tetraubiquitin chains. For each variant and condition, the dwell time distributions of 2900 – 11000 events from at least 200 single-molecule FRET efficiency traces were analyzed. Errors represent the standard errors of the exponential fits of the dwell-time distributions.

Supplemental Table 2:

Proteasome variant	Mean ± s.e.m (s)
WT proteasome	1.64 ± 0.10
WT proteasome + 10 μM K48-Ub_4	1.39 ± 0.10
ARR-Pru-ΔUIM proteasome	2.05 ± 0.18
ARR-Pru-ΔUIM proteasome + 10 μM K48-Ub_4	1.42 ± 0.14
ARR-Pru-ΔUIM-A89F proteasome	1.42 ± 0.15
ARR-Pru-ΔUIM-A89F proteasome + 10 μM K48-Ub_4	1.13 ± 0.11

Supplemental Table 2: Substrate tail-insertion and engagement kinetics. The mean values of the time constants for tail insertion and engagement of SspB-delivered I27V15P substrate were determined by fitting the dwell time distribution of the tail insertion phase in the FRET-based substrate processing assay with Gamma function for wild-type proteasome and various proteasome variants in the absence and presence of unanchored K48-linked tetraubiquitin chains.
